# Medical News and Miscellany

**Published:** 1897-01

**Authors:** 


### Medical News and Miscellany.

**Dr. R. L. Smith** has removed from Kirk to Marfa.

---

**Dr. J. Z. Ferrell** has moved from Tyler to Mt. Sylvan, and Dr. R. B. Harben from Minerva to Richardson.

---

**Dr. Sam Cunningham,** of Taylor, Texas, was elected president of the Austin District Medical Society at the late regular meeting, December 24th, ult. The society had a fine meeting that day, and enjoyed a royal banquet at the close.

---

**Dr. D. C. Jones** has removed from Waco to Cameron, Texas.

---

**Dr. J. R. McCown** has removed from Hutto to Willow City, Texas.

---

**Death of Mrs. Smith.**—It is with profound regret that the JOURNAL announces the death recently at her home in Galveston, of Mrs. Smith, wife of Professor Albert J. Smith, of the Texas Medical College. We beg to extend to Dr. Smith assurances of our deep and sincere sympathy in his sad bereavement.

---

**Dr. F. B. MacRae,** from Atlanta, Ga., has located in Temple, Tex., and comes highly recommended as a well qualified physician. He devotes much attention to abdominal surgery, and has a fine record as an operator. The doctor at once subscribed for the "Red Back," something that no up-to-date physician can afford to be without.

---

**Quarantine Appointment.**—Dr. J. C. Mayfield, formerly of Velasco, late of Galveston, has been appointed State Quarantine Officer as Galveston, vice Dr. W. F. Blunt resigned. Dr. Blunt has been in the service many years, and his retirement, on account of ill health, will be a serious loss to the service. Dr. Mayfield is a graduate of the Medical Department Tulane University, of the class of 1874.

---

**Asylum Appointments.**—At the Terrell branch of the Texas State Lunatic Asylum—the North Texas Hospital for Insane—Dr. David L. Gaillard, late first assistant physician at that institution, has been appointed superintendent, vice Dr. C. M. Rosser resigned. Dr. Gaillard was formerly of Greenville, Hunt counsy, and is a graduate of Louisville Medical College, of the class of 1877.

---

**Chattanooga Medical College.**—The JOURNAL is always pleased to note the honors won by Texas boys in the several medical colleges. At a meeting of the graduating class of 1896-7, of the Chattanooga Medical College, Mr. Lyle G. Thornton, of Texas, was elected to deliver the valedictory. The election was the result of a competitive elocutionary contest in public. Dr. Lyle will, therefore, deliver the class valedictory at the commencement, March 23, 1897. We are proud of the record made by Texas students every year.

---

**Dr. R. J. Pope,** of Jonesboro, Texas, writing, says: "Enclosed find $2.00, to renew my subscription to the "Red Back," [12th consecutive year.—Ed.] The Journal is *all* we could wish."

This means a great deal: it is a high compliment, coming, as it does, from a medical scholar, a reader and observer, capable of discriminating, and withal a man who does not speak lightly, but who weighs his words. The Journal values such an expression. Such endorsement is most grateful, and gives us encouragement to continue the good work.

---

**Death of Dr Scoggins.**—Dr. Chas. M. Scoggins, awhile located and practicing at Throckmorton, Texas; whence he removed to Graham, Texas about a year ago, had given up practice, and had entered the service of Myer Bro. Drug Co., of St. Louis, as their traveling agent and representative in Texas. He passed through Austin a short while previous to his death, and called on all the doctors, leaving samples of the preparations he was handling, and he made many friends by his courteous and genial manner. We were much shocked to read in the press dispatches, on the 22nd of December, ult., that he was found on the bank of the river, at San Antonio, in a dying condition, and evidences pointed to the fact that he had suicided by the use of morphine. He left no statement, and there is no cause assigned for the act. Dr. Scoggins seemed to be in good health and spirits while in Austin a short while before, and this announcement was a great shock to his friends. He leaves a wife, but no children, so far as we know. Dr. Scoggins was a native of Georgia, and a graduate of the Southern Medical College at Atlanta, of the class of 1886.

---

**Our Christmas Gift.**—We were not forgotten. We received a souvenir. It is a beauty. It consists of a lot of "dead heads," but they have a life expression, and are therefore a work of art. It consists of a set of six calendars, arranged into skeleton sketches; skulls, put to the body of living persons, and representing, two of them, the doctor; one as an expert witness—giving his opinion, the other an old doctor making the diagnosis at the bedside of a sick girl, and they are strikingly life-like. These water-color sketches mounted on calendars make an appropriate and acceptable office ornament. The sketches are reproductions of the originals from the brush of the celebrated artist-physician—Louis Crusius, A. M., M. D., St. Louis, and are sent us by the *Antikamnia Chemical Co.*, St. Louis, as a Christmas souvenir—as "an expression of the esteem" in which that firm "holds the medical laborer in the journalistic field." Thanks.

Mr. Venus had achieved fame; had reached the goal of his ambition; his works—articulated skeletons, were sold in every quarter of the globe. Yet—he was not happy, because the idol of his heart had declined the honor of being Mrs. Venus, saying she "did not wish to regard herself, nor yet to be regarded, in such a bony light."

We do not mind being regarded in a bony light, we are used to it—and are happy still.

---

[**Apropos** of our editorial on "Rational Medicine," in this issue, the following, extracted from an article by Dr. M. P. Overholser, in the *New York Medical Journal* of December 19, 1896, will be read with interest, and will illustrate more fully our meaning when we speak of assisting nature by reinforcing the army of occupation. It is truly wonderful what the study of bacteriology has revealed, and the end is not yet. It is within the bounds of a reasonable probability that within the first half of the 20th century, immunity against all forms of contagious disease may be secured by inoculation on the principle here discussed.—Ed.]:

"Animal life depends, in a large measure, upon the activity of its protoplasm. The more active the protoplasmic matter of its primitive units—these amœboid elements—the more vital force and vital resistance in the organism.

"The nuclein molecule of the white corpuscle is said to be the most active molecular structure of the body and its wonderful retentive of life, 'possessing marked powers of recuperation after being partly decomposed, as shown by the fact that, after being partly decomposed by salt solution, if this solution be diluted by the addition of large quantities of water the nuclein is restored to its original form.' While the function of the white corpuscle as a nutritive vitalizing agent is an all-important one, which could be dwelt upon at much greater length, yet the part it plays in the human economy as a defensive and protective agent is equally as important and essential to life."

"Let us watch these little bodies—for instance, when an irritant seeks to gain an entrance to the blood current: 'How they rush by the thousands to the breach to repel their invaders, which are clamoring to get in through a wound, by throwing up their breastworks within the gap, and how fearless and earnest is the struggle, as shown by the dead and dying of the warriors in the shape of pus corpuscles!' We call it inflammation, but it is simply an encounter between the leucocytes on the one side and the micro-organisms on the other. It is a warfare between the invaders from without and the defenders from within —a battle of the cells against the foes which threaten our physical prosperity."

"Thus, as these wandering cells float through the blood-vessels, to and fro along their numerous roads and lines, in close communication with all parts of our body, 'they play the part of an active sanitary department, with numerous corps of police stationed in all parts of our system, charged with the function of guarding their possessor against the inroads and attacks of enemies from without which constantly threaten us with disease and death. Thus, inflammation is not to be looked upon as an unnatural and diseased process, but as one which has a true physiological significance in that it is an effort on the part of the leucocytes to save us from the consequences of infection.'"

